# Hemophagocytic lymphohistiocytosis in patients with inflammatory bowel diseases: a systematic review

**DOI:** 10.3389/fimmu.2025.1575297

**Published:** 2025-08-15

**Authors:** Tingwei Lan, Qinhuan Luo, Xiaojuan Guo, Xuan Jiang

**Affiliations:** ^1^ Tsinghua University, Tsinghua Medicine, School of Medicine, Beijing, China; ^2^ Beijing Tsinghua Changgung Hospital, Department of Gastroenterology, Beijing, China

**Keywords:** hemophagocytic lymphohistiocytosis, inflammatory bowel diseases, Crohn’s disease, ulcerative colitis, hemophagocytic syndrome

## Abstract

**Introduction:**

Hemophagocytic lymphohistiocytosis (HLH) is a rare but life-threatening hyperinflammatory syndrome. Patients with inflammatory bowel diseases (IBD) appear to be at increased risk of developing HLH, potentially due to immunosuppressive therapies. However, the epidemiology, clinical characteristics, management strategies, and outcomes of HLH in this population remain poorly understood.

**Methods:**

We conducted a systematic review of the literature using PubMed, Web of Science, and Embase. A total of 97 secondary HLH (sHLH) cases and 18 HLH cases with genetic mutations were identified in IBD patients.

**Results:**

Among IBD patients, sHLH predominantly affected males with Crohn’s disease, with a median age of 33.5 years and a median disease duration of 4 years. Most patients were on thiopurines for IBD management and were in clinical remission at sHLH onset. The most common triggers were infections (particularly CMV and EBV), followed by lymphoma. The overall survival rate for sHLH was 62.5%. Most patients successfully resumed IBD maintenance therapy within 5 months after the sHLH episode, with minimal complications and rare recurrence of IBD or HLH. Older age, lymphoma-induced HLH, and lack of biologic or thiopurine therapy were potential factors associated with mortality. Compared to sHLH, primary HLH patients were younger, more frequently male, predominantly had Crohn’s disease, were less likely to be in remission despite biologic therapy, and had better outcomes with hematopoietic stem cell transplantation (HSCT).

**Discussion:**

This study provides a comprehensive characterization of HLH in IBD patients, offering valuable insights to guide future research aimed at improving clinical outcomes in this unique population.

**Systematic Review Registration:**

osf.io identifier, 10.17605/OSF.IO/5GY3E.

## 1 Introduction

Hemophagocytic lymphohistiocytosis (HLH) is a life-threatening hyperinflammatory syndrome leading to systemic inflammation and multiorgan dysfunction. It is characterized by the dysregulated immune cell function (e.g. macrophages, cytotoxic T cells), which causes the inability to properly terminate immune responses (1–3). HLH can be classified into primary (genetic) HLH, which is often linked to genetic defects in the perforin-dependent cytotoxic pathway or inflammasome activation (2, 3), and secondary HLH (sHLH), triggered by infections, malignancies, or autoimmune diseases (2). Early diagnosis and treatment are critical, as the condition carries a high mortality rate if left untreated.

Inflammatory bowel disease (IBD), encompassing Crohn’s disease (CD), ulcerative colitis (UC), and IBD-unclassified (IBD-U), is a chronic inflammatory disease of the gastrointestinal tract with a complex interplay of genetic, environmental, and immunological factors (4–6). The relationship between HLH and IBD is multifaceted. On one hand, treatments for IBD, particularly immunosuppressants and biologics, impair immune surveillance and increase the risk of opportunistic infections, lymphoma or other HLH triggers (7–9). On the other hand, genetic mutations associated with HLH, such as *XIAP* mutations, further complicate the disease course in a subset of IBD patients (10). In addition, diagnosing HLH in the context of IBD is particularly challenging, as its clinical manifestations may mimic an IBD flare, and HLH-associated cytopenia may be mistaken for the adverse effects of immunosuppressants.

Although cases of HLH in IBD patients have been increasingly reported, the current understanding of its epidemiology, clinical features, triggers, management, and outcomes remains limited. Most insights come from case reports and small case series, which highlight the diagnostic challenges and variability in clinical presentation and management.

This study aims to provide a comprehensive analysis of HLH in IBD patients by systematically reviewing the available literature. We focus on the clinical features, triggers, treatment, and outcomes of HLH in this population, as well as the disease activity and management of IBD before and after HLH. By doing so, we aim to improve the understanding and management of this complex and rare condition.

## 2 Methods

### 2.1 Search strategy

A comprehensive search of PubMed, Web of Science and Embase was performed. The search strings for each database were listed in **Supplementary Table 1**. The last search was performed on Jan 21^st^, 2025.

### 2.2 Study selection

Articles, review articles and meeting abstracts including clinical information about HLH events in IBD patients were included in this systematic review. The exclusion criteria were as follows: (1) articles not in English or Chinese, (2) articles not containing the details of individual cases (e.g., a study showing only the number of patients with IBD in a large cohort of HLH), and (3) interventional clinical trials. The HLH-2004 criteria (11) were used to define HLH. Cases with inadequate information to diagnose HLH according to the HLH-2004 criteria were excluded. Cases without adequate information to diagnose HLH but claimed to fulfill the HLH-2004 criteria were included. The records were initially screened according to the title and abstract to remove duplicates and exclude irrelevant studies. Then full texts were extracted to evaluate for the inclusion and exclusion criteria. Simultaneously, the reference lists of review articles with previously published cases were reviewed manually for further eligible articles. The protocol was pre-registered in osf.io with the DOI 10.17605/OSF.IO/5GY3E.

### 2.3 Data extraction and quality assessment

Data were collected and organized in a spreadsheet. The following data were collected for each selected article: name of the first author, publish year, country of the corresponding author, journal name, sex, age at HLH diagnosis, IBD subtype (UC, CD, or IBD-U) and Montreal classification, IBD disease duration at HLH diagnosis, IBD disease status at HLH diagnosis (remission, active, refractory or worsening), current and past IBD treatment (including 5-aminosalicylic acids (5-ASAs), steroids, immunomodulators, biologics, surgery and nutrition therapies), clinical manifestations, pathogenic factors for secondary HLH, treatment for HLH and complications, clinical outcomes (healed or died), hospital stay and IBD treatment after HLH. Finally, another duplicate check was performed to remove the cases reported repeatedly in different articles based on the information collected above. Two authors (T.L. and Q.L.) evaluated this procedure independently.

### 2.4 Statistical analysis

Descriptive data were calculated as percentages for discrete findings. Chi-square or Fisher exact tests were used for group comparisons. We considered P values less than 0.05 as statistically significant. Statistical analyses were performed using PRISM version 9.5.0 (GraphPad Software, San Diego, CA) and R version 4.1.0.

## 3 Results

### 3.1 Article selection through systematic review

The initial database search identified 6,717 records, of which 1,053 duplicates were excluded. The titles and abstracts of the remaining 5,664 records were screened based on the inclusion and exclusion criteria. A total of 232 records passed the initial screening, and their full texts were retrieved for further evaluation. During the full-text review, 138 records were excluded due to non-fulfillment of the inclusion criteria while 8 additional publications were identified through a review of the reference lists of review articles containing previously published cases. Ultimately, 115 cases from 102 publications across 30 years (1995-2024) and 27 countries were included in this study (**Supplementary Figure 1**, **Supplementary Tables 2, 3**).

### 3.2 Secondary HLH

#### 3.2.1 Basic characteristics

A total of 97 cases without reported relevant genetic mutations were identified, accounting for 84.35% of the total cases. Their basic characteristics were summarized in **Table 1**. Among the patients, 60 (61.9%) had CD, 34 (35.1%) had UC and 3 (3.1%) had IBD-U. Gender was reported for 96 cases, of which 57 (59.4%) were male. The median age at sHLH onset was 33.5 years (interquartile range [IQR]: 22-45.75 years), and the median disease duration at sHLH onset was 4 years (IQR: 2–6 years). Among the 96 patients that reported outcomes, 36 (37.5%) succumbed to sHLH.

**Table 1:** Demographics and clinical characteristics of sHLH cases stratified with IBD subtypes.

Characteristic	Total	CD	UC	IBD-U
Number of patients	97	60	34	3
Median age (IQR) (n^*^)	33.5 (22-45.75) (92)	29 (21-43) (57)	41 (29-50.5) (32)	13 (10-20) (3)
%Male (n/N)	59.4% 57/96	65.0% 39/60	48.5% 16/33	66.7% 2/3
Median disease duration (y) (IQR) (n^*^)	4 (2-6) (43)	3 (1.4975-5.75) (26)	4.5 (3-6.5) (15)	3 (2.5-3.5) (2)
%Died of HLH (n/N)	37.5% 36/96	33.9% 20/59	44.1% 15/34	33.3% 1/3

*Number of reported cases.

#### 3.2.2 Triggers of HLH

**Figure 1:**
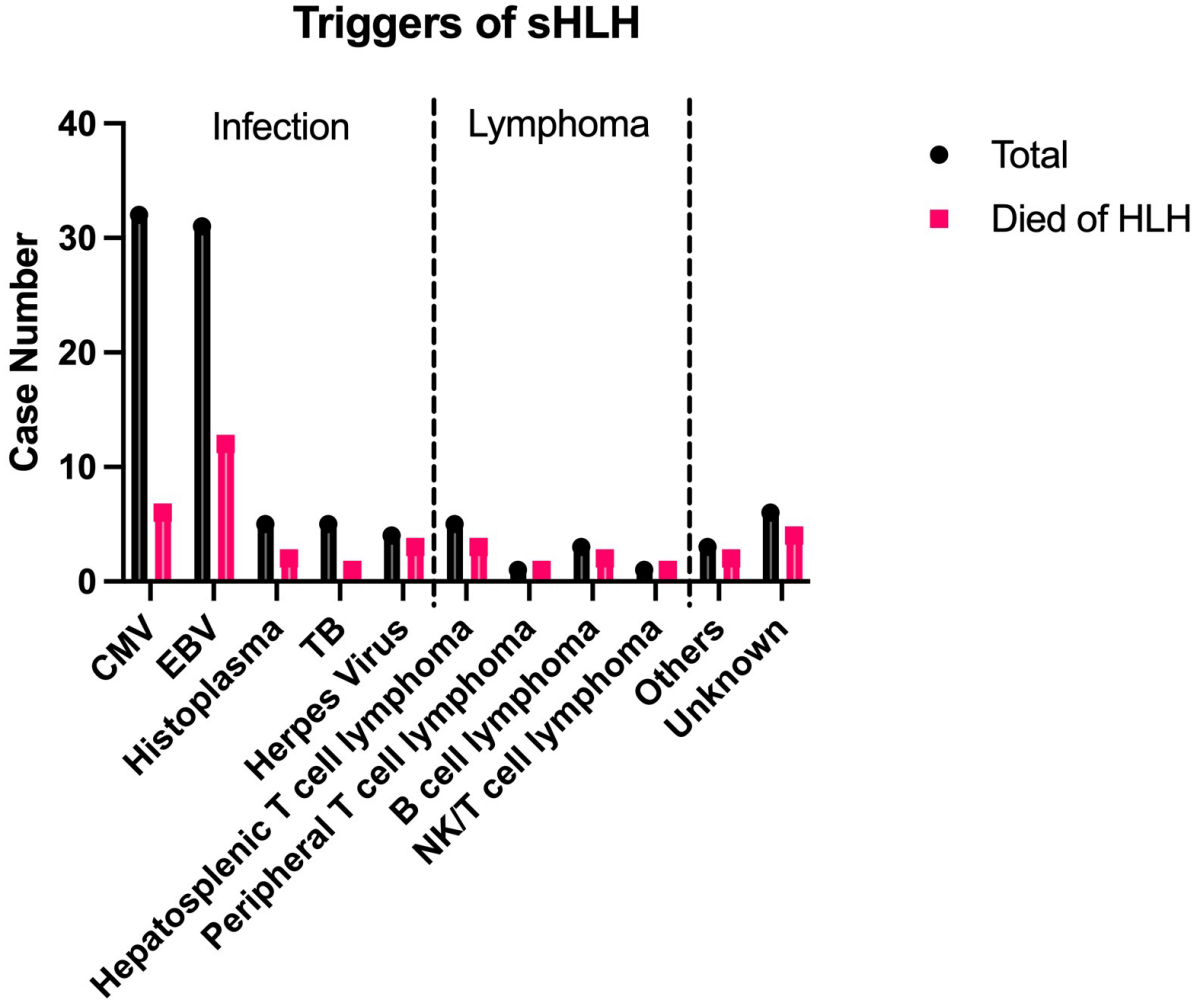
Triggers and clinical outcomes of sHLH cases.

Of the 93 cases reporting the triggers of sHLH, 6 had unknown causes. **Figure 1** shows the distribution and motality of common sHLH triggers. In 80 cases (86.0%), HLH was secondary to infections. Cytomegalovirus (CMV) (n=32, 40.0%) and *Epstein-Barr* virus (EBV) (n=31, 38.8%) were the most common pathogens implicated in sHLH among IBD patients. Other pathogens included *Histoplasma* (n=5, 6.3%), *Mycobacterium tuberculosis* (TB) (n=5, 6.3%), herpes viruses (n=4, 5.0%), parvovirus B19 (n=1), COVID-19 (n=1), *Klebsiella pneumoniae* (n=1), *Leishmania* spp. (n=1) and *Candida albicans* (n=1). Two cases involved mixed infections: one with CMV and EBV, and the other one with CMV and Histoplasma. Cases with only CMV or EBV infections and without co-existing malignancy were associated with lower mortality (n=12, 21.8%) compared with the sHLH events provoked by other infections (n=8, 44.4%).

Lymphoma was another significant cause of sHLH in IBD patients, accounting for 10.8% of cases (n=10) and associated with a poor prognosis. Among the 9 cases reporting clinical outcomes, 7 (77.8%) resulted in death due to sHLH. Hepatosplenic T-cell lymphoma (HSTCL) was the most common subtype (n=5, 50.0%) triggering sHLH in IBD patients. In 5 of the 10 cases, lymphoma was considered secondary to EBV infection.

In addition to infections and lymphoma, other factors linked to sHLH development in IBD patients included splenic artery thrombosis, COVID-19 vaccination, and liver transplantation.

#### 3.2.3 IBD status at sHLH onset

A total of 49 cases reported the IBD status at sHLH onset. 34 (69.4%) patients had well-controlled IBD at the time of HLH onset. Among patients who survived sHLH, 58.8% (n=10) patients were in remission from IBD. Among patients who succumbed to sHLH, the ratio was 75.0% (n=24).

Among the 34 cases in IBD remission, 27 had available data on gastrointestinal (GI) manifestations. Most of these cases exhibited GI symptoms at the onset of sHLH (n=20, 74.1%), including abdominal pain (n=13, 65.0%), diarrhea (n=7, 35.0%), GI bleeding (n=5, 25.0%), vomiting (n=5, 25.0%) and weight loss (n=5, 25.0%). These findings underscore the importance of distinguishing HLH from IBD relapse in clinical management. Among the 15 cases not in IBD remission at the onset of sHLH, 10 had available data on GI symptoms, all of whom presented with GI symptoms at onset. This further emphasizes the need to differentiate HLH from IBD exacerbation in patients with active IBD.

#### 3.2.4 IBD medications at sHLH onset

93 cases reported the use of IBD medication at sHLH onset (**Table 2**). At the time of sHLH onset, most of the patients were receiving thiopurines for the treatment of IBD, with most of these patients using azathioprine (AZA). 28 cases (30.1%) were receiving biologics or small molecules, including infliximab (IFX), adalimumab (ADA), ustekinumab (UST), vedolizumab (VDZ) and upadacitinib. Additionally, 3 cases were receiving no treatment for IBD at the time of sHLH onset.

**Table 2:** IBD medications at sHLH onset.

IBD medication	Case number, n (%)
Steroids	18 (19.4%)
Immunomodulators	60 (64.5%)
thiopurines	59 (63.4%)
AZA	47 (50.5%)
6-MP	11 (11.8%)
Thalidomide	1 (1.1%)
5-ASA	21 (22.6%)
mesalamine	17 (18.3%)
sulfasalazine	2 (2.2%)
Biologics and small molecules	28 (30.1%)
IFX	19 (20.4%)
ADA	4 (4.3%)
UST	2 (2.2%)
VDZ	2 (2.2%)
Upadacitinib	1 (1.1%)
No treatment	3 (3.2%)

#### 3.2.5 Clinical manifestations

**Table 3** summarized the distribution of HLH clinical manifestations in the sHLH patients with IBD. Fever (n=95), high Ferritin level (n=92, 94.8%), cytopenia (n=83, 85.6%), phagocytosis (n=72, 74.2%) and splenomegaly (n=63, 64.9%) were the most commonly reported manifestations. Notably, 21.1% (n=16) cases reported no splenomegaly and 11.1% (n=9) cases reported no phagocytosis. All the cases without phagocytosis presented with splenomegaly, liver dysfunction and high ferritin level, therefore were easy to be recognized. However, 6 of the 16 cases without splenomegaly also reported no signs of liver dysfunction (including elevated aminotransferases or bilirubin, and hepatomegaly), therefore were easy to be neglected. These 6 cases were all female with CD, presented with high ferritin level, and 5 of them had CMV infection.

**Table 3:** HLH clinical manifestations in sHLH patients with IBD.

Symptom	Presented	Not presented	Not reported
Fever	95	0	2
Splenomegaly	63	13	21
Peripheral cytopenia	83	2	12
Hypertriglyceridemia	44	17	36
Hypofibrinogenemia	32	7	58
Phagocytosis	72	9	16
Low or absent NK cell activity	16	1	80
Ferritin >500ng/mL	92	0	5
Elevated soluble CD25	32	0	65

In addition to the HLH-2004 criteria, HScore is another widely used diagnostic tool for sHLH, primarily designed for adults (12). None of the 90 sHLH cases aged ≥14 years had a reported HScore; however, 14 of these cases provided sufficient data to allow calculation. The median HScore was 272 (IQR: 237.25-297.25), with only 2 cases having HScores below the diagnostic threshold 169 (138 and 140, respectively). These findings suggest that the HScore may also be applicable for diagnosing sHLH in IBD patients.

**Table 4** summarized the other clinical manifestations in the sHLH patients with IBD. 5 cases did not report clinical manifestations beyond HLH-related signs. Among the remaining 92 cases, gastrointestinal symptoms were reported in 60 cases, including abdominal pain (n=27, 45.0%), GI bleeding (n=18, 30.0%), diarrhea (n=16, 26.7%), vomiting (n=11, 18.3%) and weight loss (n=11, 18.3%). 10 cases reported active IBD complications or extraintestinal manifestations, including joint pain (n=4), mouth ulcer (n=3), discharging perianal fistula (n=1), ileus (n=1) and toxic megacolon (n=1). 9 cases (7 with CD and 2 with UC) reported no GI symptoms at sHLH onset.

**Table 4:** Other clinical manifestations in sHLH patients with IBD.

Symptom	Case number, n (%)
GI symptoms	60 (65.2%)
Abdominal pain	27 (29.4%)
GI bleeding	18 (19.6%)
Diarrhea	16 (17.4%)
Vomiting	11 (12.0%)
Weight loss	11 (12.0%)
No GI symptoms	9 (9.8%)
Liver dysfunction	66 (71.7%)
Bilirubin elevation	19 (20.7%)
Hepatomegaly	38 (41.3%)
Aminotransferase elevation	45 (48.9%)
Respiratory symptoms	42 (45.7%)
Upper respiratory tract infections	12 (13.0%)
Dyspnea	19 (20.7%)
Cough	9 (9.8%)
Abnormal chest CT findings	23 (25.0%)
Coagulation-related symptoms	7 (7.6%)
Petechia	4 (4.4%)
Epistaxis	2 (2.2%)
DIC	1 (1.1%)
Neurologic symptoms	8 (8.7%)
Dizziness	2 (2.2%)
Seizures	2 (2.2%)
Confusion	1 (1.1%)
Face dumbness	1 (1.1%)
Hyposmia	1 (1.1%)
Retinitis	1 (1.1%)
Other symptoms	52 (56.5%)
Fatigue	24 (26.1%)
Lymphadenopathy	22 (23.9%)
Anorexia	11 (12.0%)
Rash	9 (9.8%)
Headache	9 (9.8%)
Night sweats	8 (8.7%)
Myalgia	6 (6.5%)

Liver dysfunction was observed in 66 cases, characterized by elevated aminotransferases (n=45, 68.2%), hepatomegaly (n=38, 57.6%), and elevated bilirubin levels (n=19, 28.8%). Most cases with liver dysfunction were associated with EBV infection (n=27, 40.9%), followed by CMV infection (n=20, 30.3%). Respiratory symptoms were noted in 42 cases, including upper respiratory tract infections (e.g., pharyngitis, tonsillitis) (n=12, 28.6%), dyspnea (n=19, 45.2%), cough (n=10, 23.8%), and abnormal chest CT findings (e.g., lung infiltration, pleural fluid) (n=23, 54.8%). Of these, 6 cases (14.3%) progressed to respiratory failure. EBV (n=17, 40.5%) and CMV (n=15, 35.7%) infections were the most common causes of respiratory symptoms.

Coagulation-related symptoms were reported in 6 cases, including petechia (n=4), epistaxis (n=2) and DIC (n=1). Neurologic symptoms were documented in 8 cases, including dizziness (n=2), seizures (n=2), confusion (n=1), face dumbness (n=1), hyposmia (n=1) and retinitis (n=1). Other symptoms commonly observed in HLH patients with IBD included fatigue (n=24), lymphadenopathy (n=22), anorexia (n=11), rash (n=9), headache (n=9), night sweats (n=8) and myalgia (n=6).

#### 3.2.6 Treatment of HLH secondary to infection

**Figure 2:**
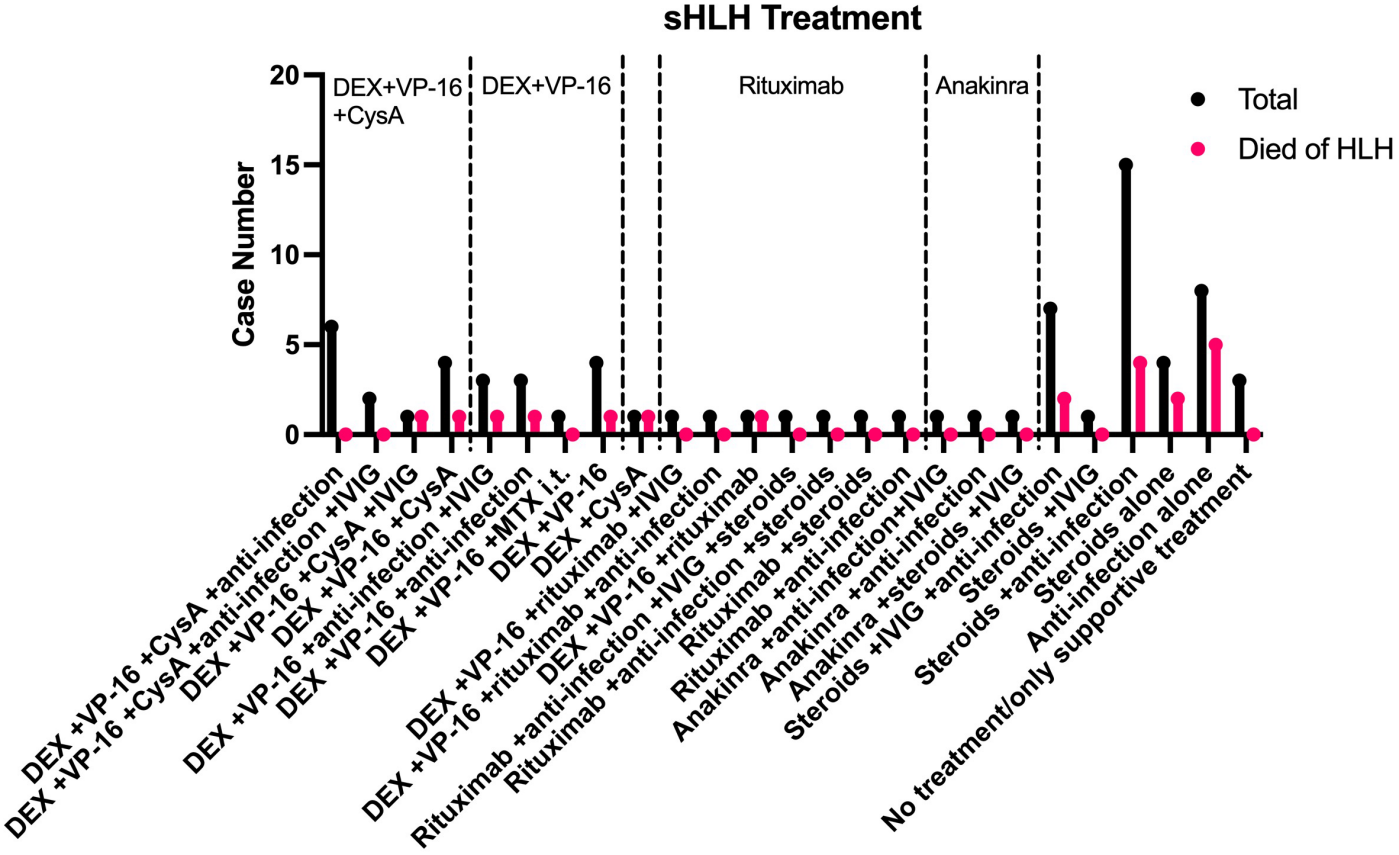
Treatments and clinical outcomes of sHLH cases.

The treatment of HLH typically involves immunomodulators, cytotoxic agents and therapies targeting the primary diseases (13). Due to the limited number of cases, here we focused on the treatment outcomes of 73 HLH cases secondary to infections without co-existing malignancy (**Figure 2**). The overall survival rate is 72.6% (n=53). Treatment details were not reported for 2 cases.

For mild HLH secondary to infection, guidelines recommend using steroids or anti-infection therapy alone to achieve remission (13, 14). Among the 73 cases, 8 received anti-infection therapy alone, and 4 received steroids alone, yielding an overall survival rate of 41.7% (n=5). Combining anti-infection therapy with steroids improved the survival rate to 73.3% (n=11).

For moderate to severe HLH, the HLH-94 protocol (15), which includes dexamethasone (DEX), etoposide (VP-16), and cyclosporin A (CysA), is recommended as first-line therapy. Among the cases treated with DEX, VP-16, CysA (± intravenous immunoglobulin (IVIG) ± anti-infection therapy), the survival rate was 84.6% (n=11). For cases receiving DEX and VP-16 (± IVIG ± anti-infection therapy ± intrathecal methotrexate [MTX]), the survival rate was 72.7% (n=8).

In recent years, rituximab, a monoclonal antibody targeting CD20, has been utilized in HLH treatment (16, 17). 7 cases treated with regimens including rituximab, 3 of which received it in combination with DEX and VP-16. The overall survival rate for cases receiving rituximab was 85.7% (n=6). Additionally, anakinra, a novel interleukin-1 receptor antagonist, has been used for HLH treatment (18). Three cases receiving anakinra all survived the HLH event, demonstrating promising outcomes.

#### 3.2.7 Post-HLH treatment of IBD

A total of 18 cases reported the management of IBD following recovery from HLH (**Table 5**). 3 cases did not initiate any IBD treatment post HLH. Of these, two reported no relapses of HLH or IBD during follow-up periods of 1 and 2 years, respectively, while the third case did not include follow-up information.

**Table 5:** IBD therapies post-HLH in sHLH patients with IBD.

IBD therapy	Case number
5-ASA	2
Low dose systemic steroids	2
Low dose systemic steroids +sulfasalazine	1
Thiopurines	2
MTX	2
IFX	4
ADA	1
No treatment	3

15 cases resumed IBD therapy after recovering from HLH. Among these, 12 had been using thiopurines for IBD remission maintenance at the time of HLH onset. Most cases avoided restarting thiopurines, with only 2 opting for thiopurine-based therapy. Neither of the 2 cases experienced HLH relapse within 1-year follow-up, though one switched from AZA to 5-ASA due to a reduction in WBC count. 3 cases retained their pre-HLH IBD regimens, using IFX, UST or low dose prednisolone combined with sulfasalazine respectively.

7 of the 18 cases provided details on follow-up duration, with a median follow-up of 15 months (IQR 12-18.5 months). Among the 18 cases, 2 reported IBD relapses during follow-up, occurring at 12 and 18 months post-HLH while under maintenance therapy with 5-ASA or low-dose prednisolone, respectively. None of the cases reported HLH relapse. However, one case using MTX for IBD remission maintenance experienced two episodes of elevated EBV-DNA levels at 13 and 18 months post-HLH, which were successfully managed with a single dose of rituximab.

The timing for restarting IBD treatment varied across cases. 7 cases specified the timing. One patient resumed UST when parvovirus B19 was undetectable. 3 patients restarted IFX at 1.25, 2, and 3 months post-HLH, respectively, without adverse events. Immunomodulators were reintroduced later than biologics: one case restarted MTX at 4 months, and another restarted 6-mercaptopurine (6-MP) at 5 months post-HLH. One patient resumed AZA at 1 month post-HLH but transitioned to 5-ASA due to WBC count reduction.

#### 3.2.8 Prognosis

A total of 29 cases reported the duration of hospitalization, with a median stay of 30 days (IQR: 17–41 days). Among the 16 cases that survived HLH, the median hospital stay was also 30 days (IQR: 18.5–36 days). For the 13 cases that succumbed to HLH, the median hospital stay was slightly shorter at 26.5 days (IQR 14-39.5).

To identify the potential risk factors for sHLH motality in IBD patients, we compare the demographics and clinical characteristics between the IBD patients survived from and succumbed to sHLH. We found that age at sHLH onset, triggers of sHLH, and current IBD medications were risk factors of sHLH motality in IBD patients in the univariate analysis (**Table 6**). For each one year increase in age, the risk of death increased by about 5% in IBD patients with sHLH (OR 1.050, 95% CI 1.021 to 1.084, *p*=0.0011). For triggers of sHLH, patients with lymphoma-induced sHLH had a higher risk of succumbing to sHLH (OR 7.259, 95% CI 1.394 to 35.61, *p*=0.0115). For current IBD medications, patients on biologics or thiopurines had a lower risk of succumbing to sHLH (OR 0.202, 95% CI 0.065 to 0.764, *p*=0.0126).

**Table 6:** Univariate analysis of risk factors for sHLH motality in IBD patients.

Risk factor	Survived (N=60)	Died (N=36)	*P* value
Age, median (IQR)	26 (20-36)	43 (32.25-54)	**0.0005**
%Male (n)	52.5% (31/59)	69.4% (25/36)	0.1337
IBD subtype, n (%) CD UC IBD-U	39 (65.0%) 19 (31.7%) 2 (3.3%)	20 (55.6%) 15 (41.7%) 1 (2.8%)	0.7225
IBD disease activity at sHLH onset %Remission, (n/N)	74.2% (23/31)	61.1% (11/18)	0.3568
Extraintestinal manifestations	1 (1.7%)	3 (8.3%)	0.1467
Trigger, n (%) Infection Lymphoma Infection +lymphoma Others Unknown or not reported	53 (88.3%) 1 (1.7%) 1 (1.7%) 1 (1.7%) 4 (6.7%)	22 (61.1%) 2 (5.6%) 5 (13.9%) 2 (5.6%) 5 (13.9%)	**0.0272**
Current IBD medication 5-ASA Steroids Thiopurines THL Biologics Steroids +5-ASA Steroids +thiopurines Thiopurines +5-ASA Biologics +thiopurines Biologics +steroids Steroids +thiopurines +5-ASA Biologics +thiopurines +steroids Biologics +steroids +5-ASA Surgery Surgery +steroids No medication Not reported	0 (0.0%) 0 (0.0%) 26 (43.3%) 0 (0.0%) 12 (21.7%) 1 (1.7%) 0 (0.0%) 4 (6.7%) 6 (10.0%) 2 (3.3%) 3 (5.0%) 0 (0.0%) 1 (1.7%) 1 (1.7%) 1 (1.7%) 1 (1.7%) 1 (1.7%)	4 (11.1%) 1 (2.8%) 7 (19.4%) 1 (2.8%) 5 (13.9%) 1 (2.8%) 3 (8.3%) 3 (8.3%) 1 (2.8%) 0 (0.0%) 4 (11.1%) 1 (2.8%) 0 (0.0%) 1 (2.8%) 0 (0.0%) 2 (5.6%) 2 (5.6%)	**0.0252**
Hospital stay, median (IQR)	30 (18.5-36)	26.5 (14-39.5)	0.7215

Values in bold denote statistical significance (p<0.05).

We further included age, sHLH triggered by lymphoma and currently on biologics or thiopurines in the multivariate logistic regression model. Cases without information about age, trigger and current IBD medications were excluded, and totally 86 cases were included in the model. All these 3 factors were still significant in the multivariate analysis (**Table 7**).

**Table 7:** Multivariate analysis of risk factors for sHLH motality in IBD patients.

Risk factor	Survived (N=53)	Died (N=33)	OR	95% CI	*P* value
Age, median (IQR)	26 (19-36)	43 (33-57)	1.052	1.018-1.091	**0.0038**
Trigger, n (%) Lymphoma	2 (3.8%)	7 (21.2%)	11.136	2.078-89.524	**0.0091**
Current IBD medication Currently on biologics or thiopurines	50 (94.3%)	22 (66.7%)	0.1374	0.0265-0.5526	**0.0085**

Values in bold denote statistical significance (p<0.05).

Most of the sHLH events in IBD patients were induced by infection. Therefore, we also performed a univariate analysis in the sHLH cases induced by infection. 75 cases were included in this analysis, and the result was summarized in **Supplementary Table 4**. Similar to the previous results, age and current IBD medications were also significant risk factors for the mortality of sHLH induced by infection in IBD patients. The pathogens of infection and anti-HLH therapies were not identified as significant risk factors. We further included age and currently on biologics or thiopurines in the multivariate logistic regression model established with sHLH cases induced by infection. Cases without information about age and current IBD medications were excluded, and totally 71 cases were included in the model. The results were summarized in **Supplementary Table 5**. Both factors were still significant in the multivariate analysis.

### 3.3 Primary HLH

Among the 115 cases included in this study, 22 cases underwent genetic tests. 18 cases identified relevant mutations. 15 cases were identified with confirmed HLH-related genetic mutations, including 13 with *XIAP* mutations, one with an *SH2D1A* mutation, and one with an *STXBP2* mutation. Gender was not reported for two cases; the remaining 13 cases were all male patients. Additionally, three cases reported mutations affecting immunoregulation, although these were not canonical HLH-related mutations: *IL-10R*, *CD40LG*, and *NOD2* and *NLRP12*, respectively. Among the 15 cases with available data on age at HLH onset, the median age was 9 years (IQR: 6–20). Of the 11 cases specifying IBD subtypes, the majority were diagnosed with CD (n=10, 90.9%). The median IBD duration at HLH onset, reported in 15 cases, was 2 years, notably shorter than that observed in patients with secondary HLH. Unlike secondary HLH cases, most patients with these mutations were not in IBD remission at HLH onset (n=14, 82.4%) and a significant proportion (n=9, 69.2%) were receiving biologics for IBD treatment. Six cases identified infections as triggers for HLH, with EBV being the most common pathogen (n=4, 66.7%), followed by CMV and methicillin-resistant *Staphylococcus aureus* (MRSA).

Clinical manifestations were reported in 13 cases. Among the clinical features in the HLH-2004 diagnostic criteria, fever (n=13, 100.0%), cytopenia (n=11, 84.6%), splenomegaly (n=10, 76.9%), elevated ferritin levels (n=9, 69.2%), and hemophagocytosis (n=6, 46.2%) were the most frequently documented features. Accompanying gastrointestinal symptoms were noted in six cases, including diarrhea (n=4, 66.7%), abdominal pain (n=2, 33.3%), hematochezia (n=2, 33.3%), and weight loss (n=1, 16.7%). One case reported an extraintestinal manifestation (oral ulcer). Liver dysfunction was also common, characterized by elevated aminotransferases (n=8, 61.5%), hepatomegaly (n=5, 38.5%), and elevated bilirubin (n=1, 7.7%). Respiratory symptoms were observed in a subset of cases, including pneumonia (n=2, 15.4%), pharyngitis (n=1, 7.7%), cough (n=1, 7.7%), and pleural fluid (n=1, 7.7%). Other commonly reported symptoms included lymphadenopathy (n=4, 30.8%) and fatigue (n=3, 23.1%).

Hematopoietic stem cell transplantation (HSCT) is considered the first-line treatment for HLH in patients with pathogenic mutations. Of the 15 cases that provided treatment details, eight underwent HSCT, achieving a survival rate of 87.5% (n=7). The overall survival rate across all cases was 76.5% (n=13).

## 4 Discussion

HLH is a life-threatening hyperinflammatory syndrome which demands prompt recognition and intervention. The inherent immune dysregulation and the widespread use of immunosuppressive therapies not only render IBD patients particularly susceptible to HLH, but also complicate the diagnosis and management of HLH in this population. In this study, we systematically reviewed 115 cases of HLH in IBD patients, focusing on their basic characteristics, triggers, treatment, outcomes, and the management of IBD before and after HLH onset. To our knowledge, this is the most extensive review of HLH in IBD patients to date. Unlike previous reviews, we adhered to the HLH-2004 diagnostic criteria and excluded cases with uncertain HLH diagnoses (19, 20). We also identified age, induced by lymphoma and currently on biologics or thiopurines as potential factors associated with the sHLH mortality of IBD patients.

In our study, 84.4% of the cases were classified as sHLH, with a male predominance (59.4%) and a CD/UC ratio of 1.76. Infection was the leading trigger of sHLH, with CMV and EBV being the most commonly implicated pathogens. Malignancies, particularly lymphomas, were also notable triggers. These findings align with a large-scale national-wide analysis (21), suggesting that the characteristics of our reviewed population closely resemble those of the broader HLH-IBD population.

Consistent the prior study (21), we identified CMV and EBV infections as the most frequent triggers of sHLH in IBD patients. Lymphomas were also remarkable triggers and were associated with poor prognosis. Importantly, in half of the lymphoma cases, EBV was a preceding factor. Previous studies demonstrated that EBV-associated HLH was often linked to thiopurines and anti-TNF agents (19, 22), and consistently, 76.3% of sHLH cases in our review involving patients on these medications. However, considering the scarcity of HLH, the necessity of screening EBV infection in IBD patients before using thiopurine or anti-TNF therapies remains controversial (23, 24). Although rarer, other pathogens, such as *Histoplasma* and *Mycobacterium tuberculosis*, were associated with a mortality rate double that of CMV or EBV-triggered HLH, likely due to delays in pathogen identification. These findings highlight the need for gastroenterologists to consider less common pathogens when managing HLH in IBD patients.

The advent of biologics and small-molecule therapies has expanded treatment options for IBD but has also been accompanied by reports of HLH. In our analysis, 17.9% (n=5) of sHLH cases in patients on biologics or small molecules involved therapies beyond anti-TNF agents, such as VDZ and UST. These findings highlight that sHLH, though uncommon, should be recognized as a possible complication in the biologics era, even among patients not treated with anti-TNF agents.

We found that among the clinical manifestations mentioned in HLH-2004 diagnostic criteria, fever, high ferritin level, cytopenia, phagocytosis and splenomegaly were the most commonly reported ones of sHLH in IBD patients. Interestingly, we identified 6 cases without splenomegaly or liver dysfunction, all of whom were female with CD and mostly had CMV infection. These findings suggest the critical role of ferritin testing in female CD patients with fever and cytopenia, even in the absence of splenomegaly or liver dysfunction, for early HLH recognition.

Our findings demonstrated that the combination of DEX, VP-16 and CysA, the 3 medications in HLH-94 protocol, achieved a high survival rate (84.6%) in infection-associated HLH. The combination of steroids, IVIG and anti-infection therapies produced comparable clinical outcomes, whereas steroids or anti-infection therapies alone were associated with poorer survival rates. These results support the adoption of more radical treatment strategies for sHLH. Moreover, regimens containing rituximab or anakinra also showed efficacy comparable to or exceeding the HLH-94 protocol. As novel agents like emapalumab (25–27) and ruxolitinib (28, 29) are increasingly utilized in HLH management, further studies are needed to evaluate their safety and efficacy in IBD-associated HLH.

IBD treatment is typically discontinued during the acute HLH phase. Few studies have explored optimal strategies and timing for resuming IBD treatment post-HLH. We identified 18 sHLH cases reporting post-HLH IBD management, 15 of which resume IBD maintenance medications. Most of them avoid thiopurines. None of them reported HLH recurrence, with only one case reported re-emergence of EBV-DNA. Two cases reported IBD relapse within 18 months while on 5-ASA or low-dose prednisone, underscoring the importance of timely resumption of appropriate IBD maintenance therapy. 4 cases reintroduced biologics within 3 months without complications, suggesting their early reinstatement may be safe. In contrast, reintroduction of immunomodulators occurred later. More robust evidence is needed to guide IBD maintenance therapy post-HLH.

We also reviewed 15 primary HLH cases as well as 3 HLH cases with mutations affecting immunoregulation. The most common mutation is XIAP mutation, which is the second most common cause of monogenic IBD (6) and typically causes recurrent HLH and refractory IBD (30). Patients with primary HLH were younger, more often male, predominantly had CD, and were rarely in remission despite biologic therapy. Compared to sHLH, these patients had better prognosis with HSCT. These findings highlight the importance of differentiating primary HLH from sHLH in young patients with refractory IBD.

This study has several limitations. Despite the similarities of basic characteristics with a large-scale national-wide study (21) and the consistency of results with prior studies, the reliance on case reports introduces inherent reporting bias. Standardized diagnostic assessments may not have been consistently applied or fully reported in some cases, potentially affecting the identification and classification of HLH events. The lack of uniformity in data reporting may also introduce bias. In addition, long-term follow-up data were unavailable for most cases. Well-designed case-control or cohort studies are needed to account for confounding factors, confirm our findings, and provide stronger evidence for the management of HLH in IBD patients.

In conclusion, HLH in IBD patients poses unique challenges requiring tailored diagnostic and therapeutic approaches. Increased awareness and further research will be pivotal in improving outcomes for this vulnerable population.

## Data Availability

The original contributions presented in the study are included in the article/**Supplementary Material**. Further inquiries can be directed to the corresponding author.
