# Supplementary material for: Hemophagocytic lymphohistiocytosis in patients with inflammatory bowel diseases: a systematic review
Source: Front Immunol. 2025 Aug 15;16:1575297. doi: 10.3389/fimmu.2025.1575297 (PMC12394226; doi:10.3389/fimmu.2025.1575297)
Supplement: Supplementary file 1 [file DataSheet1.docx]

**Supplementary Materials** (Including Supplementary Tables 1, 3-5 and Supplementary Figure 1)

**Supplementary Table 1. Search Strategies**

**PubMed**

("Crohn Disease"[Mesh] or Crohn disease[tw] or Crohn's Enteritis[tw] or Regional Enteritis[tw] or Crohn's Disease[tw] or Crohns Disease[tw] or Inflammatory Bowel Disease 1[tw] or Ileocolitis[tw] or Granulomatous Colitis[tw] or Terminal Ileitis[tw] or Regional Ileitides[tw] or Regional Ileitis[tw] or CD[tw] or “Inflammatory Bowel Diseases”[Mesh] or inflammatory bowel disease[tw] or “Colitis, Ulcerative”[Mesh] or ulcerative colitis[tw] or colitis gravis[tw] or idiopathic proctocolitis[tw] or UC[tw] or colitis ulcerosa[tw] or idiopathic ulcerative colitis[tw] or chronic ulcerative colitis[tw] or ulcerative proctitis[tw] or pancolitis[tw] or inflammatory colitis[tw] or IBD[tw])

AND

("Lymphohistiocytosis, Hemophagocytic"[Mesh] or hemophagocytic syndrome[tw] or hemophagocytic lymphohistiocytosis[tw] or macrophage activation syndrome[tw] or "Macrophage Activation Syndrome"[Mesh])

**Web of Science**

TS=(Crohn Disease OR Crohn's Enteritis OR Regional Enteritis OR Crohn's Disease OR Crohns Disease OR Inflammatory Bowel Disease 1 OR Ileocolitis OR Granulomatous Colitis OR Terminal Ileitis OR Regional Ileitides OR Regional Ileitis OR CD OR Inflammatory Bowel Diseases OR inflammatory bowel disease OR ulcerative colitis OR colitis gravis OR idiopathic proctocolitis OR UC OR colitis ulcerosa OR idiopathic ulcerative colitis OR chronic ulcerative colitis OR ulcerative proctitis OR pancolitis OR inflammatory colitis OR IBD)

AND

TS=(hemophagocytic syndrome OR hemophagocytic lymphohistiocytosis OR macrophage activation syndrome OR MAS OR HLH)

**Embase**

('crohn disease'/exp OR 'crohn disease' OR 'crohn enteritis' OR 'regional enteritis'/exp OR 'regional enteritis' OR 'crohns disease'/exp OR 'crohns disease' OR 'inflammatory bowel disease 1' OR 'ileocolitis'/exp OR ileocolitis OR 'granulomatous colitis'/exp OR 'granulomatous colitis' OR 'terminal ileitis'/exp OR 'terminal ileitis' OR 'regional ileitides' OR 'regional ileitis' OR 'cd'/exp OR cd OR 'inflammatory bowel diseases'/exp OR 'inflammatory bowel diseases' OR 'inflammatory bowel disease'/exp OR 'inflammatory bowel disease' OR 'ulcerative colitis'/exp OR 'ulcerative colitis' OR 'colitis gravis' OR 'idiopathic proctocolitis' OR uc OR 'colitis ulcerosa'/exp OR 'colitis ulcerosa' OR 'idiopathic ulcerative colitis' OR 'chronic ulcerative colitis'/exp OR 'chronic ulcerative colitis' OR 'ulcerative proctitis'/exp OR 'ulcerative proctitis' OR 'pancolitis'/exp OR pancolitis OR 'inflammatory colitis' OR ibd) AND ('hemophagocytic syndrome'/exp OR 'hemophagocytic syndrome' OR 'hemophagocytic lymphohistiocytosis'/exp OR 'hemophagocytic lymphohistiocytosis' OR 'macrophage activation syndrome'/exp OR 'macrophage activation syndrome' OR mas OR hlh)

Supplementary Table 3. References of the cases included

(1-102)

1. Posthuma EFM, Westendorp RGJ, Veer A, Kluinnelemans JC, Kluin PM, Lamers C. FATAL INFECTIOUS-MONONUCLEOSIS - A SEVERE COMPLICATION IN THE TREATMENT OF CROHNS-DISEASE WITH AZATHIOPRINE. Gut. 1995;36(2):311-3.

2. Sijpkens YWJ, Allaart CF, Thompson J, vantWout J, Kluin PM, denOttolander GJ, et al. Fever and progressive pancytopenia in a 20-year-old woman with Crohn's disease. Annals of Hematology. 1996;72(4):286-90.

3. Babu TGL, Boctor D, Davey A, Bond MC, Jacobson K. Cytomegalovirus-associated hemophagocytic syndrome in a child with Crohn disease receiving azathioprine. Journal of Pediatric Gastroenterology and Nutrition. 2004;39(4):418-21.

4. Koketsu S, Watanabe T, Hori N, Umetani N, Takazawa Y, Nagawa H. Hemophagocytic syndrome caused by fulminant ulcerative colitis and cytomegalovirus infection: Report of a case - The authors reply. Diseases of the Colon & Rectum. 2004;47(7):1254-5.

5. Hindupur S. Hemophagocytosis in hemophagocytic lymphohistiocytosis. American Journal of Hematology. 2005;80(4):299-300.

6. Francolla KA, Altman A, Sylvester FA. Hemophagocytic syndrome in an adolescent with Crohn disease receiving azathioprine and infliximab. Journal of Pediatric Gastroenterology and Nutrition. 2008;47(2):193-5.

7. Serrate C, Silva-Moreno M, Dartigues P, Poujol-Robert A, Sokol H, Gorin NC, et al. Epstein-Barr Virus-associated Lymphoproliferation Awareness in Hemophagocytic Syndrome Complicating Thiopurine Treatment for Crohn's Disease. Inflammatory Bowel Diseases. 2009;15(10):1449-51.

8. Miquel T, Bonnet DP, Leport J, Longuet P, Bletry O, Leport C. Hemophagocytic syndrome in the course of Crohn's disease: possible association with cytomegalovirus infection. Am J Gastroenterol. 2009;104(1):252.

9. Sun X-h, Zheng W-j, Zhang W, Zhao Y. A clinical analysis of hemophagocytic syndrome in autoimmune diseases. Zhonghua nei ke za zhi. 2010;49(10):836-40.

10. Fox CP, Shannon-Lowe C, Gothard P, Kishore B, Neilson J, O'Connor N, et al. Epstein-Barr virus-associated hemophagocytic lymphohistiocytosis in adults characterized by high viral genome load within circulating natural killer cells. Clin Infect Dis. 2010;51(1):66-9.

11. Duque G, Ferreira R, Figueiredo P, Sousa I, Ferreira M, Fernandes A, et al. Rare Cause of Fever in a Patient with Ulcerative Colitis. Inflammatory Bowel Diseases. 2011;17(6):E64-E5.

12. N'Guyen Y, Baumard S, Salmon JH, Lemoine L, Leveque N, Servettaz A, et al. Cytomegalovirus Associated Hemophagocytic Lymphohistiocytosis in Patients Suffering from Crohn's Disease Treated by Azathioprine: A Series of Four Cases. Inflammatory Bowel Diseases. 2011;17(9):E116-E8.

13. Gunson RN, Hague R, Gartner B, Aitken C. A case of a girl presenting with fever, cytopenia, liver dysfunction, hepatosplenomegaly, and hemophagocytosis in the bone marrow. Journal of Clinical Virology. 2011;51(1):1-3.

14. Presti MA, Costantino G, Della Torre A, Belvedere A, Cascio A, Fries W. Severe CMV-related pneumonia complicated by the hemophagocytic lymphohistiocytic (HLH) syndrome in quiescent Crohn's colitis: harmful cure? Inflamm Bowel Dis. 2011;17(11):E145-6.

15. Salado CT, Gallego AG, Carnerero EL, De la Cruz Ramírez D, Justiniano JM, Galán JL, et al. Hemophagocytic lymphohistiocytosis in Crohn's disease associated with primary infection by Epstein-Barr virus. Inflamm Bowel Dis. 2011;17(11):E143-4.

16. Altaf S, Atreaga GM, Joshi AY, Rodriguez V. Diffuse large B-cell lymphoma in an adolescent female presenting with Epstein-Barr virus-driven hemophagocytic lymphohistiocytosis: a case report. Journal of medical case reports. 2012;6:141-.

17. Munoz J, Shareef N, Donthireddy V. Cytomegalovirus-induced haemophagocytic lymphohistiocytosis syndrome. BMJ Case Rep. 2012;2012.

18. Hernandez-Camba A, Lakhwani S, Ramos L, Maria Raya J, Quintero E. Cytomegalovirus-associated hemophagocytic syndrome in a patient with Crohn's disease receiving azathioprine. Journal of Gastrointestinal and Liver Diseases. 2013;22(4):471-2.

19. Mun JI, Shin SJ, Yu BH, Koo JH, Kim DH, Lee KM, et al. A case of hemophagocytic syndrome in a patient with fulminant ulcerative colitis superinfected by cytomegalovirus. Korean Journal of Internal Medicine. 2013;28(3):352-5.

20. Fitzgerald MP, Armstrong L, Hague R, Russell RK. A case of EBV driven haemophagocytic lymphohistiocytosis complicating a teenage Crohn's disease patient on azathioprine, successfully treated with rituximab. Journal of Crohns & Colitis. 2013;7(4):314-7.

21. Siminas S, Caswell M, Kenny SE. Hemophagocytic lymphohistiocytosis mimicking surgical symptoms and complications: Lessons learned from four cases. Journal of Pediatric Surgery. 2013;48(7):1514-9.

22. Virdis F, Tacci S, Messina F, Varcada M. Hemophagocytic lymphohistiocytosis caused by primary Epstein-Barr virus in patient with Crohn's disease. World journal of gastrointestinal surgery. 2013;5(11):306-8.

23. Weinkove R, Dickson M, Eliadou E, Stace NH, Goossens L, Ferguson P. Fever and pancytopenia in a patient with Crohn's disease. Gut. 2013;62(9):1327-+.

24. Kukunoor S, Shivamurthy P, Mutneja R. Hemophagocytic syndrome and acute liver failure caused by herpes simplex virus. American Journal of Respiratory and Critical Care Medicine. 2014;189.

25. Javaid AI, Hahn KJ, Kwok RM, Albugeaey M, Rangnekar AS, Satoskar RS. A fatal cause of fever and pancytopenia in a patient with ulcerative colitis treated with azathioprine. American Journal of Gastroenterology. 2015;110:S389.

26. Pop CS, Becheanu G, Calagiu D, Antea P-V, Radulescu DM, Pariza G, et al. A rare complication of CMV infection in Crohn's disease - hemophagocytic syndrome: a case report. Romanian Journal of Morphology and Embryology. 2015;56(4):1535-40.

27. Popeskou S, Gavillet M, Demartines N, Christoforidis D. Hemophagocytic Lymphohistiocytosis and Gastrointestinal Bleeding: What a Surgeon Should Know. Case reports in surgery. 2015;2015:745848-.

28. Sanchez Y, Trigo C, Marquez C, Leo E, Marquez JL. Hemophagocytic lymphohistiocytosis in Crohn's Disease associated to citomegalovirus (CMV) or Epstein-Barr virus (EBV). Journal of Crohns & Colitis. 2015;9:S128-S.

29. Varma A, Honhar M, Idowu M. Hemophagocytic Lymphohistiocytosis in Ucelerative Colitis. American Journal of Gastroenterology. 2015;110:S187-S.

30. Thompson G, Pepperell D, Lawrence I, McGettigan BD. Crohn's disease complicated by Epstein-Barr virus-driven haemophagocytic lymphohistiocytosis successfully treated with rituximab. BMJ Case Rep. 2017;2017.

31. Vikse J, Cacic DL, Carlsen A, Cooper TJ, Grimstad T. A man in his 30s with ulcerative colitis and pancytopenia. Tidsskrift for den Norske laegeforening : tidsskrift for praktisk medicin, ny raekke. 2016;136(11):1010-4.

32. Dalsania R, Mandel B, Christie J. The Not so Innocent Kissing Disease: A Case of HLH in a Crohn's Patient on a Thiopurine. American Journal of Gastroenterology. 2016;111:S820-S.

33. Kalyan Sundaram A, Reed M. A rare case of CMV-induced hemophagocytic lymphohistiocytosis. Critical Care Medicine. 2016;44(12):505.

34. Ma C, Fedorak RN, Halloran BP. Recurrent Fevers After Infliximab Therapy for Ulcerative Colitis Hemophagocytic Lymphohistiocytosis. Gastroenterology. 2016;150(1):E1-E2.

35. Krause ML, Matteson EL, Messerli A. Macrophage activation syndrome in a 38 year-old woman with ulcerative colitis receiving infliximab. Journal of General Internal Medicine. 2016;31(2):S668-S9.

36. Divithotawela C, Garrett P, Westall G, Bhaskar B, Tol M, Chambers DC. Successful treatment of cytomegalovirus associated hemophagocytic lymphohistiocytosis with the interleukin 1 inhibitor - anakinra. Respirol Case Rep. 2016;4(1):4-6.

37. Beck A, Mullally J, Agonafer EP, Trifan A. Tb or not TB? The drama unfolds with macrophage activation syndrome (MAS). Journal of General Internal Medicine. 2017;32(2):S609.

38. Vakkalagadda CV, Cadena-Semanate R, Non LR. Cytomegalovirus-Associated Hemophagocytic Syndrome in a 59-Year-Old Woman with Ulcerative Colitis. American Journal of Medicine. 2017;130(7):E305-E6.

39. Miechowiecki J, Stainer W, Wallner G, Tuppy H, Aichinger W, Prammer W, et al. Severe complication during remission of Crohn's disease: hemophagocytic lymphohistiocytosis due to acute cytomegalovirus infection. Zeitschrift Fur Gastroenterologie. 2018;56(3):259-63.

40. Lucey O, Carroll I, Bjorn T, Millar M. Reactivation of latent histoplasma and disseminated cytomegalovirus in a returning traveller with ulcerative colitis. JMM Case Reports. 2018;5(12).

41. Miyaguchi K, Yamaoka M, Tsuzuki Y, Ashitani K, Ohgo H, Miyagawa Y, et al. Epstein-Barr virus-associated hemophagocytic syndrome in a patient with ulcerative colitis during treatment with azathioprine: A case report and review of literature. World Journal of Clinical Cases. 2018;6(14):776-80.

42. Roussel B, Carter V, Ekekezie C, Argueta E, Kelly CR, Jankowich MD. Hemophagocytic Lymphohistiocytosis in a Patient With Crohn's Disease. American Journal of Gastroenterology. 2018;113:S1181-S.

43. Saez-Gonzalez E, Salavert M, Cerrillo E, Moret I, Iborra M, Nos P, et al. Secondary Haemophagocytic Syndrome and Overlapping Immune Reconstitution Syndrome: Life-Threatening Complications of Anti-TNF-α Treatment for Crohn's Disease. American Journal of Gastroenterology. 2019;114(1):177-9.

44. Goetgebuer RL, van der Woude CJ, de Ridder L, Doukas M, de Vries AC. Clinical and endoscopic complications of Epstein-Barr virus in inflammatory bowel disease: an illustrative case series. International Journal of Colorectal Disease. 2019;34(5):923-6.

45. Cockbain BC, Mora Peris B, Abbara A, So CW, Cooke G. Disseminated CMV infection and HLH in a patient with well-controlled HIV and ulcerative colitis. BMJ case reports. 2019;12(2).

46. Martinez-Pillado M, Varela-Duran M, Said-Criado I, Diaz-Parada P, Rodriguez-Losada M, Mendoza-Pintos M. Disseminated tuberculosis and hemophagocytic syndrome although TB prophylaxis in patients with inflammatory bowel disease treated with Infliximab. Idcases. 2019;16.

47. Ammar H, Azouzi A, Fathallah N, Boujelben MA, Ouni B, Boussarsar M, et al. Fatal sulfasalazine-induced DRESS complicated by HHV-6 reactivation and hemophagocytic lymphohistiocytosis. European Journal of Clinical Pharmacology. 2020;76(3):467-8.

48. Brambilla B, Barbosa AM, Scholze CdS, Riva F, Freitas L, Balbinot RA, et al. Hemophagocytic Lymphohistiocytosis and Inflammatory Bowel Disease: Case Report and Systematic Review. Inflammatory intestinal diseases. 2020;5(2):49-58.

49. Tang T, Sharma V, Carlson B, Hoang L, Massey H, Brumand A, et al. Hemophagocytic Lymphohistiocytosis (HLH) Induced by Acute Epstein-Barr Virus (EBV) Infection in a Patient With Crohn's Disease on Vedolizumab. American Journal of Gastroenterology. 2020;115:S1190-S.

50. Bachmann J, Le Thi G, Brückner A, Kalteis AL, Schwerd T, Koletzko S, et al. Epstein-Barr Virus Prevalence at Diagnosis and Seroconversion during Follow-Up in Pediatric Inflammatory Bowel Disease. J Clin Med. 2021;10(21).

51. Perše B, Mataija M, Katavić M, Kardum-Skelin I, Ćuk MC, Žaja O. Hemophagocytic lymphohistiocytosis in a 13-year old girl with Crohn's disease. Archives of Disease in Childhood. 2021;106(SUPPL 2):A108.

52. Rolsdorph L, Mosevoll KA, Helgeland L, Reikvam H. Concomitant Hemophagocytic Lymphohistiocytosis and Cytomegalovirus Disease: A Case Based Systemic Review. Front Med (Lausanne). 2022;9:819465.

53. Debels L, Reynders M, Cauwelier B, Willandt B, Selleslag D, Snauwaert C. Parvovirus B19-triggered hemophagocytic lymphohistiocytosis in a patient with Crohn's disease. Acta Gastro-Enterologica Belgica. 2022;85(3):522-4.

54. Kharmach O, Lagdali N, Benelbarhdadi I, Borahma M, Ajana F-Z. Macrophage activation syndrome during Crohn's disease: a case report. Pan African Medical Journal. 2022;42.

55. Vega JET, Torrijos YMS, De Juan CJ. HEMOPHGOCYTIC LYMPHOHISTIOCYTOSIS IN CROHN'S DISEASE ASSOCIATED TO CITOMEGALOVIRUS (CMV) OR EPSTEINBARR VIRUS (EBV). European Journal of Case Reports in Internal Medicine. 2022;9:414-5.

56. Zhong Q, Ordaya EE, Fernandez SD, Lescalleet K, Larson D, Pritt B, et al. Disseminated histoplasmosis and hemophagocytic lymphohistiocytosis in a patient receiving TNF-alpha inhibitor therapy. IDCases. 2022;29.

57. Ali S, Choo S, Hosking L, Smith A, Hughes T. A case of T-cell-Epstein-Barr virus-haemophagocytic lymphohistiocytosis and sustained remission following ruxolitinib therapy. Clinical & Translational Immunology. 2023;12(7).

58. Estabrooks KL, De Silva K, Survela L, Stevenson WS. Haemophagocytic lymphohistiocytosis due to leishmaniasis following anti-tumour necrosis factor-alpha therapy. British Journal of Haematology. 2023;203(4):497-8.

59. Hean RV, Sheffield DA, Herbert K, Brewster D. Haemophagocytic lymphohistiocytosis secondary to disseminated tuberculosis in a young adult with Crohn's disease. Medical Journal of Australia. 2023;219(8):350-2.

60. Khan S, Rizvi TA, Sadiq W, Sattar SBA, Maroun R. Severe COVID-19-Induced Hemophagocytic Lymphohistiocytosis. Cureus. 2023;15(1):e34022.

61. Abu Rached N, Gambichler T, Ocker L, Schultheis B, Susok L, Schmidt W, et al. Upadacitinib treatment associated with varicella zoster infection complicated by haemophagocytic lymphohistiocytosis in a patient with severe hidradenitis suppurativa. Journal of the European Academy of Dermatology and Venereology. 2024;38(2):e139-e41.

62. Cammann VL, Tresch A, Himmelmann A, Sommerstein R, Bächli E. Fatal Hemophagocytic Lymphohistiocytosis triggered by Epstein-Barr Virus in a patient with immunosuppression. Swiss Medical Weekly. 2024;154:110S-1S.

63. Pedicelli A, Michel RP, Krassakopoulos N. Cytomegalovirus-Induced Hemophagocytic Lymphohistiocytosis in an Immunocompromised Patient with Inflammatory Bowel Disease. Case Reports in Hematology. 2024;2024.

64. Zhang JL, Yuan; Ding, Shigang; Huang, Yonghui; Zhou, Liya. 克罗恩病合并噬血细胞综合征一例并文献复习. Chinese Journal of Internal Medicine. 2013;52(9):762-4.

65. A case of Crohn’s disease with hemophagocytic syndrome [Internet]. 2022.

66. Kanaji S, Okuma K, Tokumitsu Y, Yoshizawa S, Nakamura M, Niho Y. Hemophagocytic syndrome associated with fulminant ulcerative colitis and presumed acute pancreatitis. American Journal of Gastroenterology. 1998;93(10):1956-9.

67. Bosman G, Langemeijer SM, Hebeda KM, Raemaekers JM, Pickkers P, van der Velden WJ. The role of rituximab in a case of EBV-related lymphoproliferative disease presenting with haemophagocytosis. Neth J Med. 2009;67(8):364-5.

68. Uslu N, Demir H, Balta G, Saltik-Temizel IN, Ozen H, Gürakan F, et al. Hemophagocytic syndrome in a child with severe Crohn's disease and familial Mediterranean fever. J Crohns Colitis. 2010;4(3):341-4.

69. Deneau M, Wallentine J, Guthery S, O'Gorman M, Bohnsack J, Fluchel M, et al. Natural Killer Cell Lymphoma in a Pediatric Patient With Inflammatory Bowel Disease. Pediatrics. 2010;126(4):E977-E81.

70. Côté-Daigneault J, Bernard EJ. Hepatosplenic lymphoma presenting initially as hemophagocytic syndrome in a 21-year-old man with Crohn's disease: a case report and literature review. Can J Gastroenterol. 2011;25(8):417-8.

71. Fries W, Cottone M, Cascio A. Systematic review: macrophage activation syndrome in inflammatory bowel disease. Alimentary Pharmacology & Therapeutics. 2013;37(11):1033-45.

72. Khurana A, Kowlgi NG, Kukunoor S. Hemophagocytic lymphohistiocytosis and splenic artery thrombosis: A rare association. Critical Care Medicine. 2013;41(12):A308.

73. Yellu M, Medlin S. Secondary Hemophagocytic Lymphohistiocytosis As a Presentation For Peripheral T-Cell Lymphoma. Blood. 2013;122(21).

74. Rodriguez LB, Ritchie EK. A Case Series of Adult Secondary Hemophagocytic Lymphohistiocytosis Treated at Weill Cornell Medical College. Blood. 2016;128(22).

75. Hanoun M, Dührsen U. The Maze of Diagnosing Hemophagocytic Lymphohistiocytosis: Single-Center Experience of a Series of 6 Clinical Cases. Oncology (Switzerland). 2017;92(3):173-8.

76. Arslan F, Alp S, Büyükasık Y, Ozkan MC, Şahin F, Basaran S, et al. Hemophagocytic lymphohistiocytosis in adults: Low incidence of primary neoplasm as a trigger in a case series from Turkey. Mediterranean Journal of Hematology and Infectious Diseases. 2018;10(1).

77. Booth AL, Osehobo P, Rodgers-Soriano D, Lalarukh A, Eltorky MA, Stevenson HL. Hemophagocytic Lymphohistiocytosis Secondary to Unknown Underlying Hodgkin Lymphoma Presenting with a Cholestatic Pattern of Liver Injury. Case reports in gastroenterology. 2018;12(1):99-108.

78. Galeano NF, Rivera-Penera MT, Bonilla MA, Menell JS, Kosinski M, Gupta P, et al. Transitory Hemophagocytic Lymphohistiocytosis (HLH)/Macrophage Activation Syndrome (MAS) in a Child With Very Early Onset (VEO) of Ulcerative Colitis (UC) Complicated With Primary Sclerosing Cholangitis (PSC). American Journal of Gastroenterology. 2019;114:S1383-S.

79. Tong QJ, Godbole MM, Biniwale N, Jamshed S. An Elusive Diagnosis: Case Reports of Secondary Hemophagocytic Lymphohistiocytosis and Review of Current Literature. Cureus Journal of Medical Science. 2019;11(4).

80. Amin R, Garcia-Rodriguez V, Gheeya J, Mai B, Idowu M, Mathew R, et al. Primary hepatic t-cell lymphoma with secondary hlh masquerading as acute liver failure. American Journal of Gastroenterology. 2020;115(SUPPL):S1271-S2.

81. Bashir H, Alzubi J, Awada H, Adroja S, Lane J. HEMOPHAGOCYTIC LYMPHOHISTIOCYTOSIS COMPLICATED BY ARDS AND MULTIORGAN FAILURE. Chest. 2020;158(4):A993-A4.

82. Thaker S, Pajot G, Lakhoo K, Agrawal R, Ghoulam E, Chan C. Hemophagocytic lymphohistiocytosis in a liver transplant recipient. American Journal of Gastroenterology. 2021;116(SUPPL):S1190-S1.

83. Davenport L, Chin-Hon J, Chung J, Chen XJC, Schneider J. 6-Mercaptopurine and Macrophage Activation Syndrome Case Report and Review of the Literature. Journal of Pharmacy Practice. 2023;36(4):1015-9.

84. Banz M, Stallmach A, Gassler N, Schulze PC, Fritzenwanger M, Cornely O, et al. Fatal pulmonary hemorrhage, pneumothorax and skin necrosis caused by IRIS to an <i>Aspergillus flavus</i> infection in a young patient with metamizole associated agranulocytosis. Infection. 2024;52(2):685-90.

85. Gutierrez-Rios L, Vayreda E, Calafat M, Manosa M, Domenech E, Canete F. Hepatosplenic T-cell lymphoma and inflammatory bowel disease. Revista Espanola De Enfermedades Digestivas. 2024;116(3):175-.

86. Rodrigues CM, Carvalho AC, Ventura S, Domingues Â P, Silva A, Ministro P. Persistent Fever after COVID-19 Vaccination in a Patient with Ulcerative Colitis: A Call for Attention. GE Port J Gastroenterol. 2024;31(2):129-35.

87. N'Guyen Y, Andreoletti L, Patey M, Lecoq-Lafon C, Cornillet P, Leon A, et al. Fatal Epstein-Barr Virus Primo Infection in a 25-Year-Old Man Treated with Azathioprine for Crohn's Disease. Journal of Clinical Microbiology. 2009;47(4):1252-4.

88. Lekbua A, Ouahed J, O'Connell AE, Kahn SA, Goldsmith JD, Imamura T, et al. Risk-factors Associated With Poor Outcomes in VEO-IBD Secondary to XIAP Deficiency: A Case Report and Literature Review. Journal of Pediatric Gastroenterology and Nutrition. 2019;69(1):E13-E8.

89. Coughlan T, Tee HW, Choo S, Bosco J. X-linked lymphoproliferative syndrome type 2 diagnosed in a 20 year-old male presenting with haemophagocytic lymphohistiocytosis on a background of infantile-onset crohn's disease. Internal Medicine Journal. 2014;44:25.

90. Sunseri WM, Kugathasan S, Keljo DJ, Greer JB, Ranganathan S, Cross RK, et al. IBD LIVE Case Series-Case 3: Very Early-Onset Inflammatory Bowel Disease: When Genetic Testing Proves Beneficial. Inflammatory Bowel Diseases. 2015;21(12):2958-68.

91. Ogashiwa T, Kunisaki R, Yasuhara H, Tsuda S, Koh R, Yazawa K, et al. Clinical, morphological and histological features of X-linked lymphoproliferative syndrome type 2 enteritis diagnosed in three cases with childhood onset inflammatory bowel disease. Journal of Crohn's and Colitis. 2015;9:S143.

92. Beşer ÖF, Conde CD, Kutlu T, Çullu Çokuǧraş F, Boztuǧ K, Erkan T. Inflammatory bowel disease with lethal disease course caused by a nonsense mutation in BIRC4 encoding X-linked inhibitor of apoptosis protein (XIAP). Journal of Pediatric Gastroenterology and Nutrition. 2016;62(5):e41-e3.

93. Ono S, Okano T, Hoshino A, Yanagimachi M, Hamamoto K, Nakazawa Y, et al. Hematopoietic Stem Cell Transplantation for XIAP Deficiency in Japan. Journal of Clinical Immunology. 2017;37(1):85-91.

94. Xu LJ, Luo YY, Yu JD, Lou JG, Fang YH, Chen J. X-linked inhibitor of apoptosis deficiency manifested as Crohn's disease: a case report and literature review. Zhonghua er ke za zhi = Chinese journal of pediatrics. 2018;56(1):43-7.

95. Parackova Z, Milota T, Vrabcova P, Smetanova J, Svaton M, Freiberger T, et al. Novel XIAP mutation causing enhanced spontaneous apoptosis and disturbed NOD2 signalling in a patient with atypical adult-onset Crohn’s disease. Cell Death and Disease. 2020;11(6).

96. Abdrabou S, Toita N, Ichihara S, Tozawa Y, Takahashi M, Fujiwara SI, et al. Absent X-linked inhibitor of apoptosis protein expression in T cell blasts and causal mutations including non-coding deletion. Pediatr Int. 2022;64(1):e14892.

97. He C, Li H, Zhou P, Zhang W, Li J, Li M. A novel XIAP mutation in an adult Chinese patient with refractory Crohn's disease. Clinics and Research in Hepatology and Gastroenterology. 2022;46(4).

98. Fujikawa H, Shimizu H, Nambu R, Takeuchi I, Matsui T, Sakamoto K, et al. Monogenic inflammatory bowel disease with<i> STXBP2</i> mutations is not resolved by hematopoietic stem cell transplantation but can be alleviated via immunosuppressive drug therapy. Clinical Immunology. 2023;246.

99. Maccari ME, Tron C, Speckmann C, Grp HHS. JAKi Salvage Therapy Followed by Curative Cord Blood Transplantation in a XIAP-Deficient Infant with Relapsing HLH. Journal of Clinical Immunology. 2023;43(6):1178-81.

100. Truyens M, Hoste L, Geldof J, Hoorens A, Haerynck F, Huis In ’T Veld D, et al. Successful treatment of ulcerative colitis with anakinra: a case report. Acta Gastro-Enterologica Belgica. 2023;86(4):573-6.

101. Qiu KY, Liao XY, Wu RH, Huang K, Fang JP, Zhou DH. X-linked Hyper-IgM Syndrome: A Phenotype of Crohn's Disease with Hemophagocytic Lymphohistiocytosis. Pediatric Hematology and Oncology. 2017;34(8):428-34.

102. Khojah A, Gunderman L, Bukhari A, Schutt M, Cohran V. Early-onset Crohn's disease, IgA nephropathy, and hemophagocytic lymphohistiocytosis in a patient with IL-10 receptor deficiency. Clinical Immunology Communications. 2022.

Supplementary Table 4. Univariate analysis of risk factors for motality of sHLH induced by infection in IBD patients.

|  | **Survived**  **(N=53)** | **Died**  **(N=22)** | ***P* value** |
| --- | --- | --- | --- |
| **Age, median (IQR)** | 27 (19-36) | 43.5 (33.5-56.75) | **0.0013** |
| **%Male (n)** | 54.7% (29) | 72.7% (16) | 0.1978 |
| **IBD subtype, n (%)**  CD  UC  IBD-U | 39 (73.6%)  12 (22.6%)  2 (3.8%) | 12 (54.6%)  10 (45.5%)  0 (0.0%) | 0.1099 |
| **IBD disease activity at sHLH onset**  %Remission, (n/N) | 75.9% (22/29) | 72.7% (8/11) | >0.9999 |
| **Trigger, n (%)**  CMV  EBV  Histoplasmosis  TB  Herpes virus  Mixed infections  Others | 25 (47.2%)  17 (32.1%)  3 (5.7%)  4 (7.6%)  1 (1.9%)  1 (1.9%)  2 (3.8%) | 4 (18.2%)  8 (36.4%)  1 (4.6%)  1 (4.6%)  3 (13.6%)  1 (4.6%)  4 (18.2%) | 0.0580 |
| **Current IBD medication**  5-ASA  Steroids  Thiopurines  THL  Biologics  Steroids +5-ASA  Steroids +thiopurines  Thiopurines +5-ASA  Biologics +thiopurines  Biologics +steroids  Steroids +thiopurines +5-ASA  Biologics +steroids +5-ASA  Surgery  Surgery +steroids  No medication  Not reported | 0 (0.0%)  0 (0.0%)  24 (45.3%)  0 (0.0%)  11 (20.8%)  0 (0.0%)  0 (0.0%)  4 (7.6%)  5 (9.4%)  2 (3.8%)  3 (5.7%)  1 (1.9%)  1 (1.9%)  1 (1.9%)  1 (1.9%)  0 | 4 (19.1%)  1 (4.8%)  3 (14.3%)  1 (4.8%)  2 (9.5%)  1 (4.8%)  1 (4.8%)  3 (14.3%)  0 (0.0%)  0 (0.0%)  2 (9.5%)  0 (0.0%)  1 (4.8%)  0 (0.0%)  1 (4.8%)  1 | **0.0049** |
| **Anti-HLH Therapies**  No medications beyond steroids, anti-infection drugs and supportive treatment | 19 (35.9%) | 11 (55.0%) | 0.1841 |

Supplementary Table 5. Multivariate analysis of risk factors for motality of sHLH induced by infection in IBD patients.

|  | **Survived**  **(N=49)** | **Died**  **(N=22)** | **OR** | **95% CI** | ***P* value** |
| --- | --- | --- | --- | --- | --- |
| **Age, median (IQR)** | 27 (19-36) | 43.5 (33.5-56.75) | 1.048 | 1.010-1.092 | **0.0161** |
| **Current IBD medication** Currently on biologics or thiopurines | 46  (94.9%) | 12  (54.6%) | 0.1069 | 0.0202-0.4435 | **0.0036** |

Supplementary Figure 1. Flow chart of study inclusion
